# Editorial: Gastrointestinal Hormones

**DOI:** 10.3389/fendo.2019.00498

**Published:** 2019-07-25

**Authors:** Silvano Paternoster, Damien Keating, Marco Falasca

**Affiliations:** ^1^Metabolic Signalling Group, School of Biomedical Sciences, Curtin Health Innovation Research Institute, Curtin University, Perth, WA, Australia; ^2^Molecular and Cellular Physiology Laboratory, College of Medicine and Public Health, Flinders University, Adelaide, SA, Australia

**Keywords:** glucagon-like peptides, gastrointestinal hormone, G protein coupled receptor (GPCR), NAFLD, enteroendocrine cell

The gastrointestinal tract is a vast organ hosting a broad gamut of hormone secreting cells. After more than a century since the first description in 1906 of metabolically active gut extracts by Moore (1), we are still unveiling novel functions in health and disease for this complex endocrine organ.

In the current Research Topic, we picture our current understanding of the enteroendocrine cell system (EECs), with new layers of complexity, covering not only the physiology and pathophysiology of the most well-characterized gut peptides Glucagon-like peptide-1 (GLP-1) and the Gastric insulinotropic peptide (GIP), but also many other mediators of central metabolism.

In this issues Sun et al. discuss the most recently recognized physiological impact of different gut hormones including GIP, GLP-1, Oxyntomodulin, peptide YY (PYY), serotonin, ghrelin, and the less studied insulin-like peptide 5 (INSL-5). The first four, known primarily for their anorexigenic, satiety-inducing action, while the last two for their orexigenic, appetite-inducing activity, orchestrate our metabolic response to food, maintaining our glucose, and energy homeostasis. The role of serotonin (5-HT) is also highlighted by Beyder, as a key mediator of gut-mechanosensation.

Recent studies have expanded our understanding of the physiology of different gut hormones, namely GLP-1, GLP-2, GIP, and PYY in bone metabolism. Schiellerup et al. discusses the most recent evidence updating us on the potential development of drugs based on these peptides for the management of diseases affecting bone metabolism, such as osteoporosis.

In two other reviews, an updated picture of the gut chemo-sensation of sweet and bitter tastants is dissected. Kreuch et al. delves deeper into the most recent findings surrounding the ability to detect dietary sugars, considering the recent rise in ingested non-caloric sweeteners and their impact on enteroendocrine cells and the microbiome, ultimately affecting satiety and glycaemia. Influence of this complex gut-brain axis holds a yet untapped potential for the management of chronic metabolic diseases such as obesity and diabetes. Similarly, understanding of the bitter sensation in the gut, highlighted by Xie et al., has important clinical implications. The chemo-sensation of bitter molecules beyond the tongue is transduced by different G-protein coupled receptors, namely different species of type 2 receptors (T2Rs) expressed by the enteroendocrine cells. This powerful gut axis is another tool with therapeutical implications for the modulation of different gut hormones, including the anorexigenic GLP-1, PYY, cholecystokinin (CCK), and the orexigenic Ghrelin.

Nonetheless, among all the gut-derived hormones, GLP-1 is the only one, which biology is currently exploited for the treatment of metabolic pathologies such as type 2 diabetes and obesity. Rowlands et al. expand upon the implications of GLP-1 mimicking drugs, consider the molecular targets in both acute and chronic settings, and dissect the tissue-specific downstream signaling pathways behind the recognized cardiometabolic benefits of these therapeutics.

Drugs activating the GLP-1 receptor are also beneficial in the management of another current epidemic, namely non-alcoholic fatty liver disease (NAFLD). Seghieri et al. delves deeper into this topic by reviewing the clinical benefits of molecules designed to activate both GLP-1 and Glucagon receptors, with superior anti-NAFLD properties. Nevertheless, the physiology and pathophysiology of GLP-1 is indeed much more complex than initially thought, with GLP-1 now thought to not only be acting systemically as a hormone through the bloodstream. Our understanding of gut-derived, and pancreas derived GLP-1, are both analyzed and repurposed by Paternoster and Falasca drawing more attention on the tissue-specific action of different GLP-1 species, including the once thought inactive, and much more abundant, metabolites.

Rehfeld encourages us to broaden the old concept of gut hormones inducing the release of pancreatic hormones, or incretins, currently epitomized mainly by GLP-1 and GIP. The currently recognized physiological axis linking the gut to the pancreas for whole-body metabolic control sees hundreds of different peptides, often with overlapping functions and possibly synergistic actions of yet unknown biology and metabolic impact.

A better understanding of the physiology of EECs and their involvement in a variety of physiological pathways and pathophysiological phenomenon will likely provide a strong platform for future therapeutics designed to address the morbidity of chronic metabolic diseases such as obesity and type 2 diabetes.

## Author Contributions

All authors listed have made a substantial, direct and intellectual contribution to the work, and approved it for publication.

### Conflict of Interest Statement

The authors declare that the research was conducted in the absence of any commercial or financial relationships that could be construed as a potential conflict of interest.

## References

[1] Moore B. On the treatment of Diabetus mellitus by acid extract of Duodenal Mucous Membrane. Biochem J. (1906) 1:28–38. 10.1042/bj0010028 16742013 PMC1276119

